# Combined molnupiravir-nirmatrelvir treatment improves the inhibitory effect on SARS-CoV-2 in macaques

**DOI:** 10.1172/jci.insight.166485

**Published:** 2023-02-22

**Authors:** Kyle Rosenke, Matt C. Lewis, Friederike Feldmann, Eric Bohrnsen, Benjamin Schwarz, Atsushi Okumura, W. Forrest Bohler, Julie Callison, Carl Shaia, Catharine M. Bosio, Jamie Lovaglio, Greg Saturday, Michael A. Jarvis, Heinz Feldmann

**Affiliations:** 1 Laboratory of Virology,; 2 Rocky Mountain Veterinary Branch, and; 3 Laboratory of Bacteriology, Division of Intramural Research, National Institute of Allergy and Infectious Diseases, NIH, Hamilton, Montana, USA.; 4 University of Plymouth, Plymouth, Devon, United Kingdom.; 5 The Vaccine Group Ltd, Plymouth, Devon, United Kingdom.

**Keywords:** COVID-19, Drug therapy

## Abstract

The periodic emergence of SARS-CoV-2 variants of concern (VOCs) with unpredictable clinical severity and ability to escape preexisting immunity emphasizes the continued need for antiviral interventions. Two small molecule inhibitors, molnupiravir (MK-4482), a nucleoside analog, and nirmatrelvir (PF-07321332), a 3C-like protease inhibitor, have recently been approved as monotherapy for use in high-risk patients with COVID-19. As preclinical data are only available for rodent and ferret models, here we assessed the efficacy of MK-4482 and PF-07321332 alone and in combination against infection with the SARS-CoV-2 Delta VOC in the rhesus macaque COVID-19 model. Macaques were infected with the SARS-CoV-2 Delta variant and treated with vehicle, MK-4482, PF-07321332, or a combination of MK-4482 and PF-07321332. Clinical exams were performed at 1, 2, and 4 days postinfection to assess disease and virological parameters. Notably, use of MK-4482 and PF-07321332 in combination improved the individual inhibitory effect of both drugs, resulting in milder disease progression, stronger reduction of virus shedding from mucosal tissues of the upper respiratory tract, stronger reduction of viral replication in the lower respiratory tract, and reduced lung pathology. Our data strongly indicate superiority of combined MK-4482 and PF-07321332 treatment of SARS-CoV-2 infections as demonstrated in the closest COVID-19 surrogate model of human infection.

## Graphical abstract

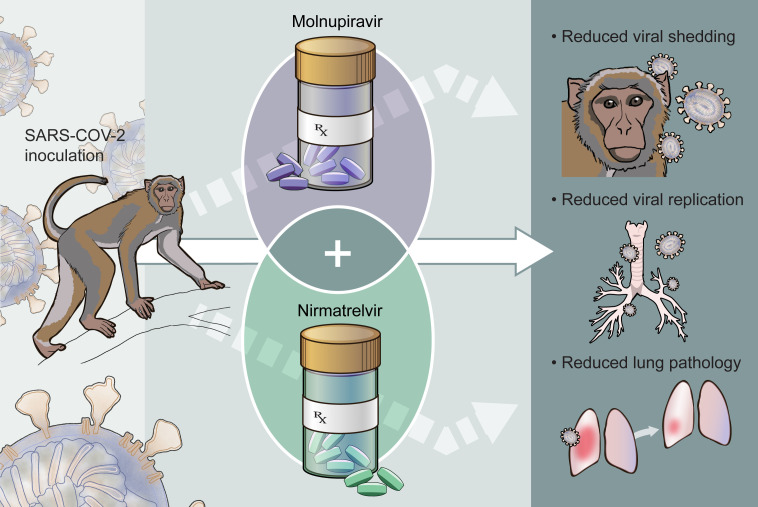

## Introduction

SARS-CoV-2, the causative agent of COVID-19 (1), was initially reported in China in 2019 (2) and has subsequently spread around the world. The pandemic is now being driven by regional waves of newly emerging SARS-CoV-2 variants. These variants emerge on a steady but unpredictable basis. To date, 13 variants of concern (VOCs) or of interest associated with mutations that alter key aspects of transmissibility, immune evasion, or severity of disease have emerged, excluding the Omicron subvariants currently circulating (3, 4). Mutations associated with VOCs are most frequently located within the spike protein, where they affect functions such as receptor binding and antibody neutralization, but can be located throughout the entire SARS-CoV-2 genome (3–9).

Molnupiravir (MK-4482) and nirmatrelvir (PF-07321332) are orally administered antivirals that have recently been approved to treat high-risk patients with COVID-19 (10–13). These compounds target distinct stages of the SARS-CoV-2 replication cycle, but neither directly targets the viral spike protein (14, 15). MK-4482 is a nucleoside analog affecting SARS-CoV-2 polymerase fidelity, resulting in mutations that ultimately reduce virus infectivity (15). PF-07321332 is a 3C-like protease inhibitor that prevents cleavage of the SARS-CoV-2 polyprotein, ultimately inhibiting viral replication (14). PF-07321332 is currently used in combination with low-dose ritonavir, an inhibitor of cytochrome P450-3A4, thus increasing the PF-07321332 serum half-life, allowing for increased potency (14).

Results from recent studies in tissue culture and rodent and ferret models suggest that MK-4482 and PF-07321332 retain activity when used as monotherapies against emerging VOCs (16–18). We investigated the efficacy of MK-4482 and PF-07321332, individually and in combination, against the SARS-CoV-2 Delta VOC in the rhesus macaque COVID-19 model (19). Notably, use of MK-4482 and PF-07321332 in combination improved the individual inhibitory effect of both drugs on clinical outcome, viral RNA, and infectious viral loads in the upper and lower respiratory tract of infected animals. Our study demonstrates efficacy of MK-4482 and PF-07321332 as monotherapy and, importantly, improved efficacy when used in combination against SARS-CoV-2 in the closest COVID-19 surrogate model of human infection.

## Results

### Study design.

**Figure 1:**
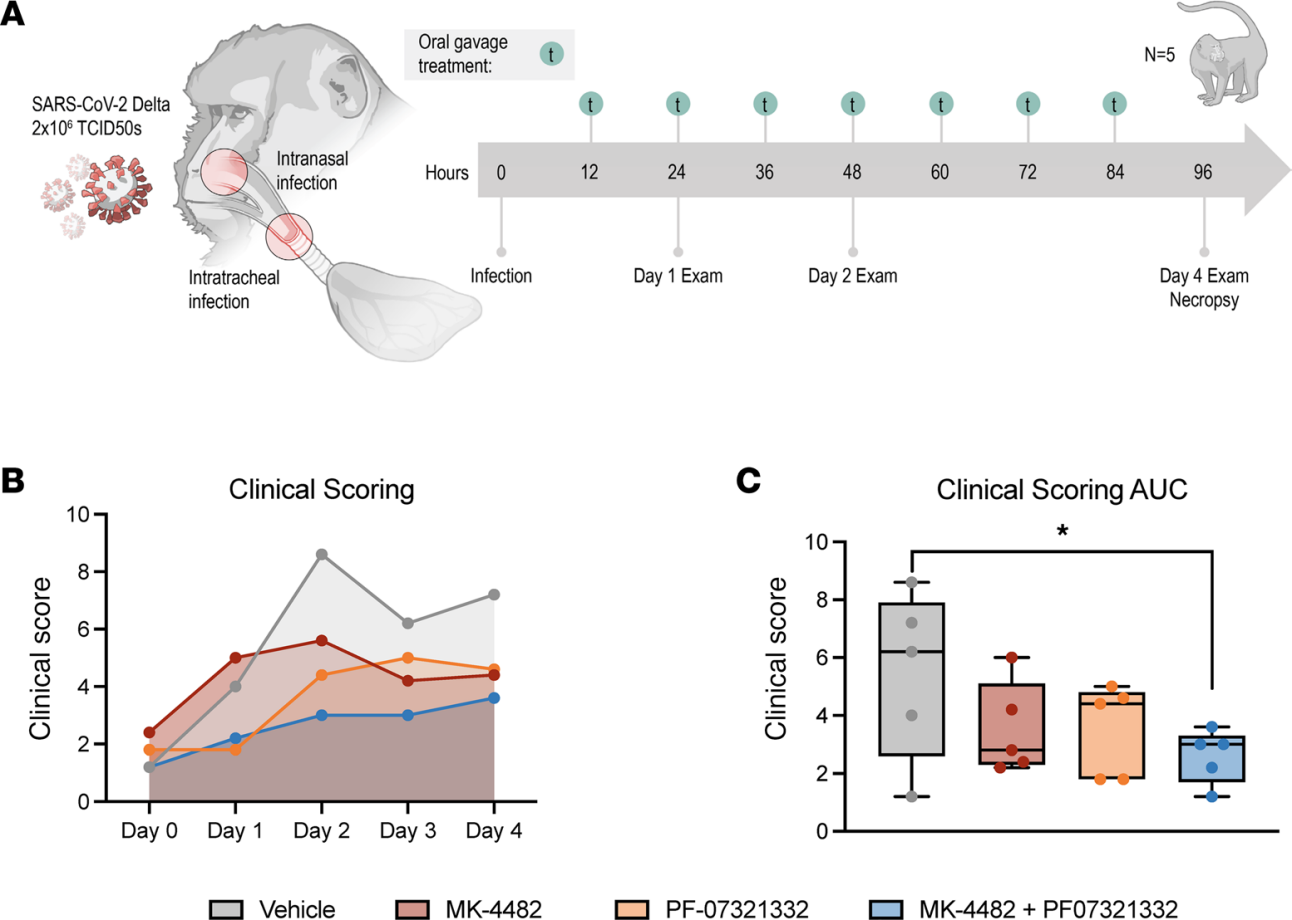
Combination therapy of MK-4482 and PF-07321332 reduces clinical scores in rhesus macaques. Experimental design (**A**). Rhesus macaques (*n* = 5) were infected with 2 × 10^6^ TCID_50_ SARS-CoV-2 by combined intranasal and intratracheal routes. Treatments were started 12 hours postinfection, with continued dosing every 12 hours. Clinical exams were conducted on 1, 2, and 4 dpi, and animals were euthanized and necropsied on 4 dpi. Clinical scoring (**B**). Animals were scored daily for clinical signs of disease over the course of the study. AUC analysis of clinical scoring (**C**). Clinical scores were calculated for each animal per day; AUC was then calculated over the course of the study and displayed in minimum-to-maximum box plot with median displayed. Ordinary 1-way ANOVA with Dunnett’s multiple comparisons test, single pooled variance, were used to evaluate significance (*P value = 0.01 to 0.05). Nonsignificant results are not indicated.

Rhesus macaques were randomly divided into vehicle, MK-4482, PF-07321332, or combination treatment groups (*n* = 5 per group). Animals were infected with 2 × 10^6^ tissue culture infectious dose 50% (TCID_50_) of SARS-CoV-2 Delta VOC by combined intranasal and intratracheal routes. Drug treatments began 12 hours postinfection with animals receiving vehicle, 130 mg/kg MK-4482 (260 mg/kg/d), 20 mg/kg PF-073211332 + 6.5 mg/kg ritonavir (40 mg/kg/d, 13 mg/kg/d), or a combination of all 3 compounds consisting of the same doses as for individual treatments. Treatments were administered by oral gavage every 12 hours (7 total treatments). The study ended 4 days postinfection (dpi) (12 hours following last treatment), at which time animals were euthanized for tissue collection (Figure 1A).

### Combination treatment reduces SARS-CoV-2–associated clinical disease.

Animals were scored for signs of disease daily by the same person blinded to the study groups using a previously established scoring sheet (19). A score of 0–15 was assigned for general appearance, skin and fur, nose/mouth/eyes/head, respiration, feces and urine, food intake, and locomotor activity. Groups scored evenly in the morning prior to inoculation (0 dpi). Animals in the vehicle group showed the highest scores throughout the experiment, peaking at 2 dpi. Animals in the group receiving the combination treatment scored the lowest throughout (Figure 1B). Animals in the groups treated with either MK-4482 or PF-07321332 alone scored in between (Figure 1B). Although differences between groups were not statistically significant on any single day, area under the curve (AUC) analysis showed a significant difference between the vehicle group and the group receiving the combination therapy (Figure 1C).

### Combination treatment reduces virus shedding.

**Figure 2:**
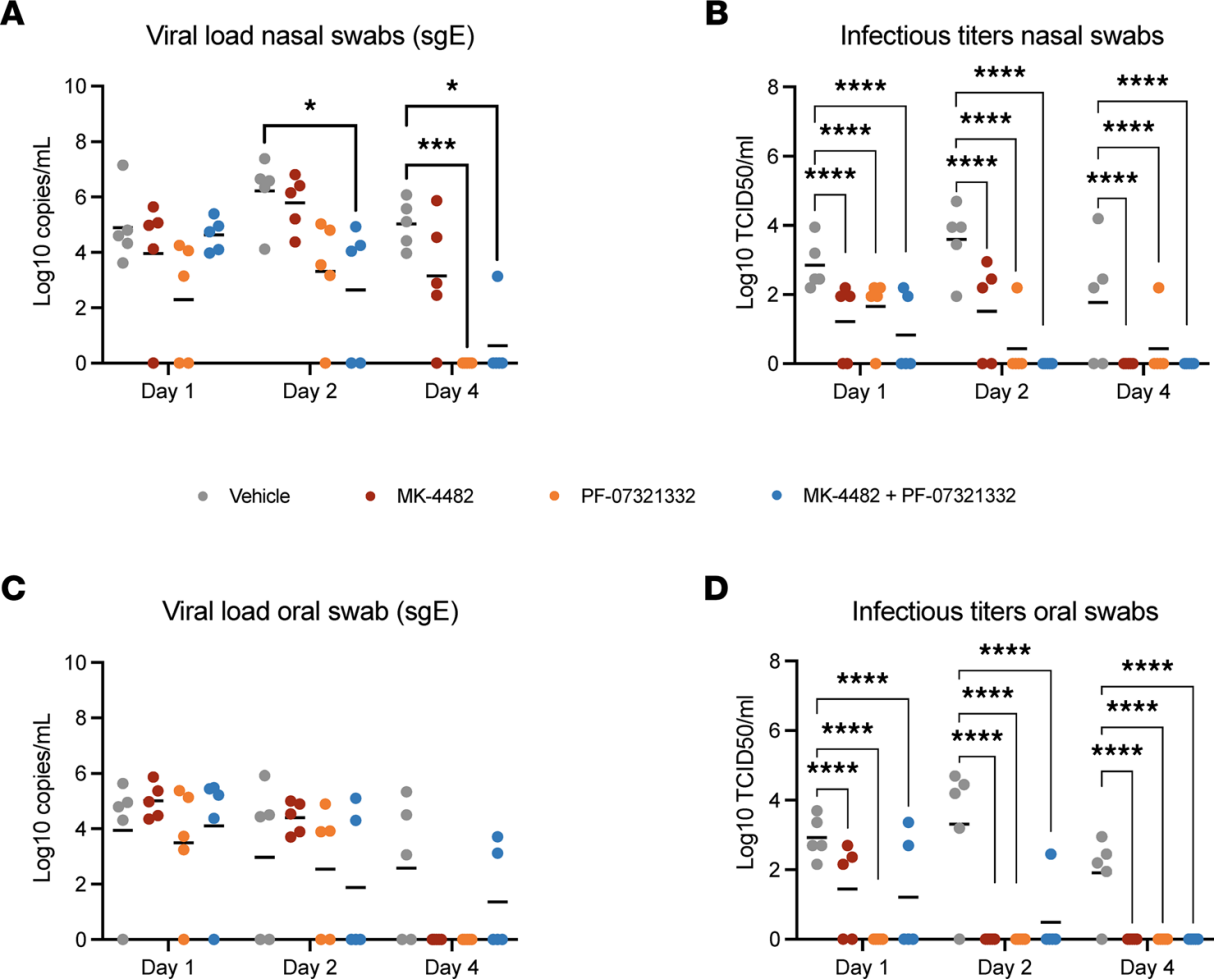
Combination therapy of MK-4482 and PF-07321332 significantly reduces virus replication in the upper respiratory tract of SARS-CoV-2–infected rhesus macaques. Viral RNA load in nasal (**A**) and oral swabs (**C**). Nasal and oral swabs (*n* = 5) were collected on 1, 2, and 4 dpi, and viral RNA loads were determined by quantitative RT-PCR targeting sgE RNA. Infectious titers in nasal (**B**) and oral (**D**) swabs. Nasal and oral swabs (*n* = 5) were collected on 1, 2, and 4 dpi, and infectivity was determined by using a TCID assay with virus titers presented as TCID_50_/mL. Data represent mean. Statistical differences in viral load and infectious virus titers in each study arm were assessed by a 2-way ANOVA using Tukey’s multiple comparisons test (*P* values, * = 0.01 to 0.05, *** = 0.0001 to 0.001, **** = <0.0001). Nonsignificant results are not indicated.

Nasal and oral swabs were collected on 1, 2, and 4 dpi. Subgenomic E–based reverse transcription PCR (sgE-based RT-PCR) was used as an initial measure of active SARS-CoV-2 replication (20). Compared with the vehicle group, sgE viral RNA loads in the nasal swabs were lower in all treatment groups and remained significantly lower in the combination therapy group for the duration of the study. The PF-07321332, but not MK-4482, monotherapy treatment group’s viral load was also significantly lower at 4 dpi (Figure 2A). These differences were further amplified in the infectious titers recovered from the nasal swabs. At 1 dpi, all treated animals had significantly lower infectious titers. By 2 dpi, the differences between treated and untreated animals were increased, and a larger subset of animals in each treatment group had no detectable infectious virus (Figure 2B). There were no significant differences in the levels of viral RNA detected in the oral swabs at any time point examined (Figure 2C), but AUC analysis performed for the entirety of the study revealed a significant difference between the combination therapy and the vehicle control (Supplemental Figure 1A; supplemental material available online with this article; https://doi.org/10.1172/jci.insight.166485DS1). Infectious virus was significantly lower at 1 dpi in all treated groups when compared with the vehicle group, and treated groups remained significantly lower over the entirety of the study (Figure 1D).

### Combination treatment reduces virus replication in the lower respiratory tract.

**Figure 3:**
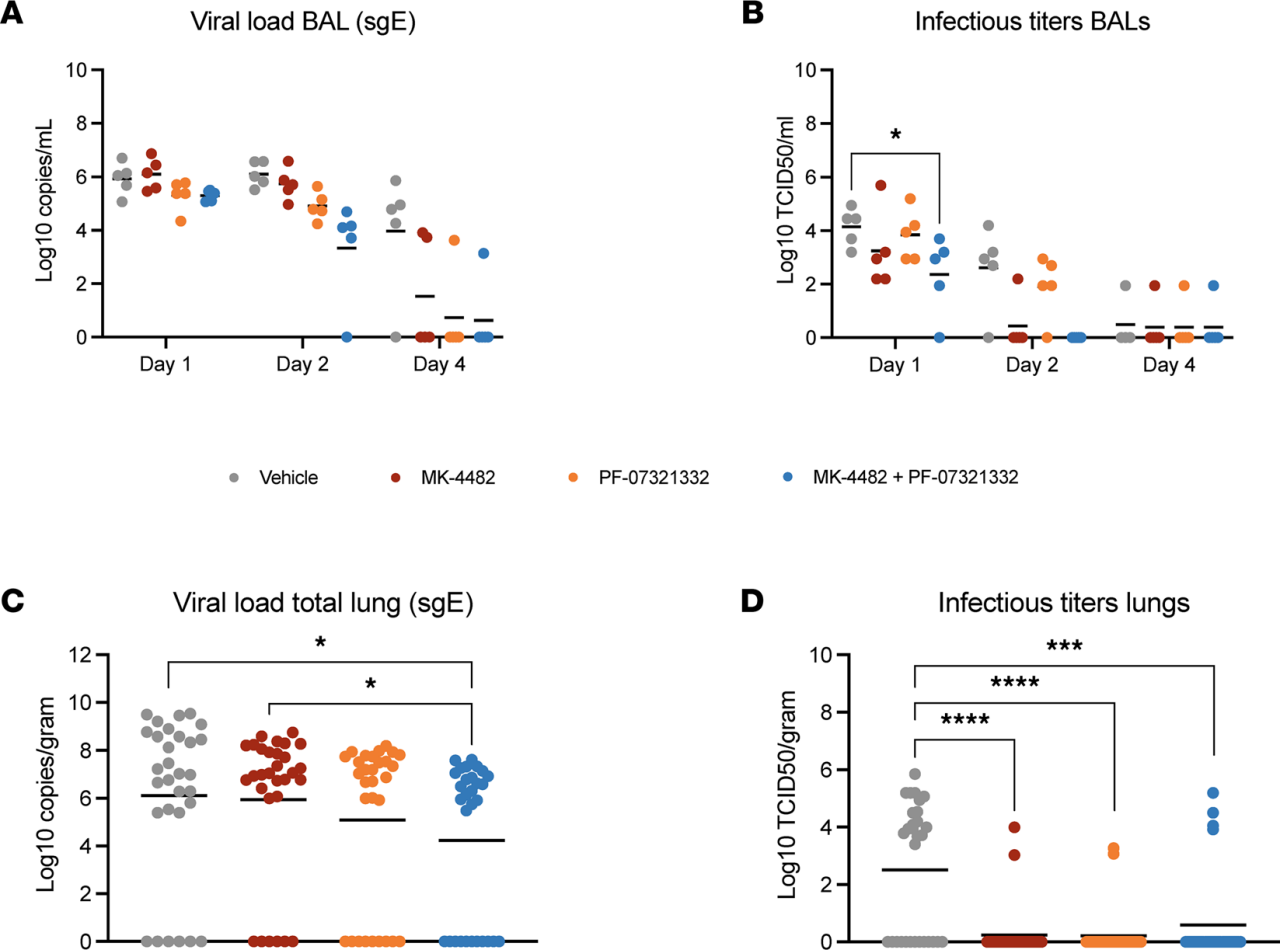
Combination therapy of MK-4482 and PF-07321332 significantly reduces viral replication in the lower respiratory tract of SARS-CoV-2–infected rhesus macaques. Viral RNA load in BAL (**A**) and total lung lobes (**C**). BAL samples (*n* = 5) were collected on 1, 2, and 4 dpi, and lung samples (*n* = 30) were collected following necropsy on 4 dpi. Viral RNA loads from BAL and lung samples were determined by quantitative RT-PCR targeting sgE RNA. Infectious titers in BAL (**B**) and total lung lobes (**D**). BAL samples (*n* = 5) were collected on 1, 2, and 4 dpi, and lung samples were collected following necropsy on 4 dpi. Infectivity was determined using a TCID_50_ assay and is presented as TCID_50_/mL. Samples from each lung lobe (*n* = 6) of each animal were assessed individually and compiled for analysis (total *n* = 30) (**C** and **D**). Data represent mean. Statistical differences in viral load and infectious virus titers in the BAL were assessed by 2-way ANOVA using Tukey’s multiple comparisons test. Statistical differences in total lung lobes were analyzed with Kruskal-Wallis with Dunn’s multiple comparisons test (*P* values, * = 0.01 to 0.05, *** = 0.0001 to 0.001, **** = <0.0001). Nonsignificant results are not indicated.

Bronchoalveolar lavages (BALs) were collected at 1, 2, and 4 dpi. sgE RNA loads were lower in PF-07321332 and combination therapy groups at 1 dpi and 2 dpi, compared with the vehicle controls (Figure 3A). The differences were more pronounced between groups at 2 dpi, but at no time was the sgE significantly different between groups (Figure 3A). AUC analysis of the BALs did reveal a significant difference between the vehicle- and combination-treated groups (Supplemental Figure 1B). Infectious virus in the BAL samples was lower in all treatment groups at 1 dpi and 2 dpi as compared with the vehicle group, a result that was statistically significantly for the combined treatment group at 1 dpi (Figure 3B). Although only 1 animal in the MK-4482 group and no animals in the combination therapy groups had detectable infectious virus at 2 dpi, these results were not statistically significant. By 4 dpi all treatment and the vehicle groups had only a single animal with detectable levels of infectious virus (Figure 3B).

Tissue samples from each lung lobe were collected at 4 dpi and RNA was isolated. Each lobe value was then pooled for comparisons between groups. Animals receiving PF-07321332 and the combination therapy had lower levels of sgE RNA viral loads in the lungs than the vehicle group; the combination therapy group had significantly less detectable sgE viral load than either vehicle- or MK-4482–treated groups (Figure 3C). Infectious virus loads in lungs were significantly reduced in all treatment groups by study end (Figure 3D).

### Drug treatments reduce pathology and viral antigen in respiratory tissues.

**Figure 4:**
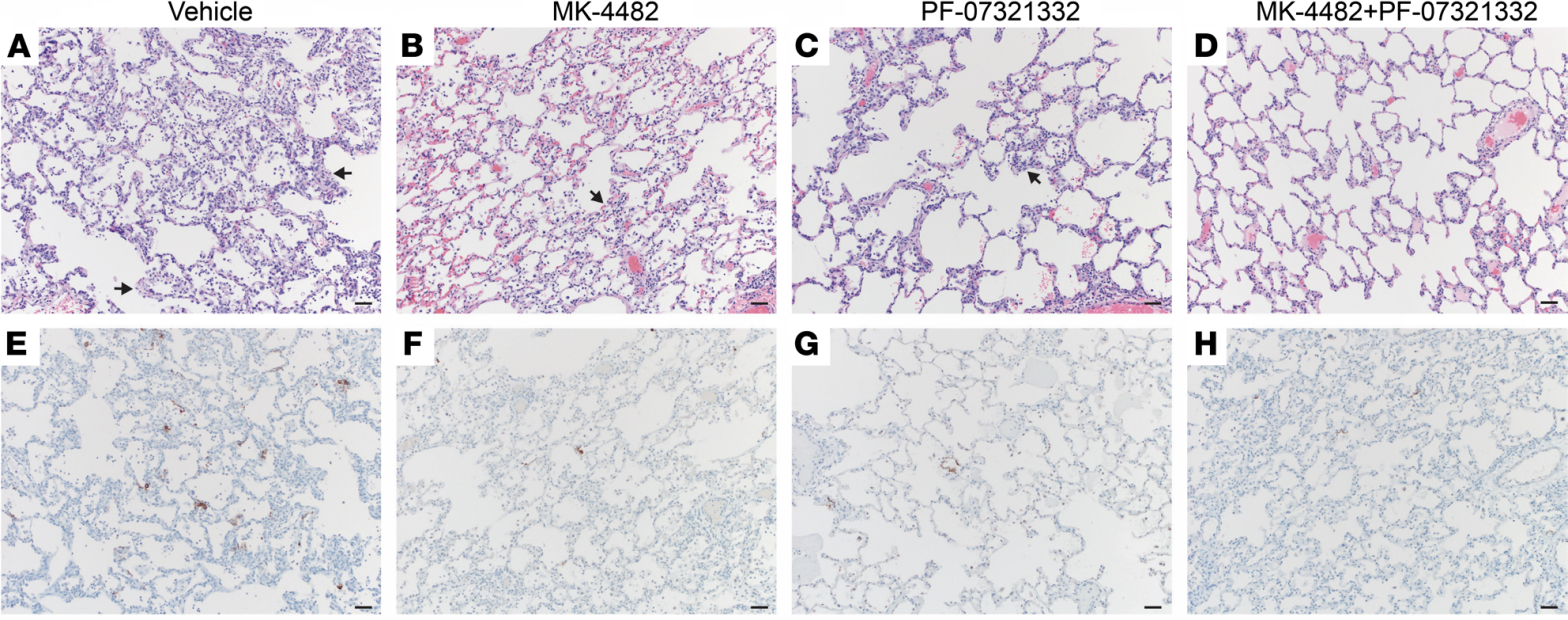
Combination therapy reduces lung pathology and SARS-CoV-2 antigen load in SARS-CoV-2–infected rhesus macaques. Lung tissues were collected on 4 dpi and stained with either hematoxylin and eosin (H&E) or immunohistochemistry (IHC). H&E staining of representative tissue sections of the lungs (**A**–**D**). IHC staining of SARS-CoV-2 antigen in corresponding representative lung sections (**E**–**H**). (**A**) Vehicle control animals showed moderate interstitial pneumonia (arrows) and moderate immunoreactivity of type I pneumocytes in 4 of 5 animals (**A** and **E**). MK-4482–treated animals showed mild interstitial pneumonia (arrows) and scattered immunoreactive type I pneumocytes in 3 of 5 animals (**B** and **F**). PF-07321332–treated animals showed mild interstitial pneumonia (arrows) and scattered immunoreactive type I pneumocytes in 2 of 5 animals (**C** and **G**). Combination treatment animals basically showed no interstitial pneumonia, with 1 of 5 animals displaying minimal lesions and scattered immunoreactive type I pneumocytes (**D** and **H**). Original magnification, 100×; scale bar = 100 μm.

Histological analysis revealed treatments decreased lung pathology. The vehicle controls developed minimal to marked pathology in 4 of 5 animals. These lesions were characterized as multifocal, mild to marked interstitial pneumonia characterized by thickening of alveolar septa by edema fluid and fibrin, small to moderate numbers of macrophages, and fewer neutrophils. Alveoli contained increased numbers of macrophages and neutrophils. Multifocal perivascular infiltrates of small to moderate numbers of lymphocytes forming perivascular cuffs were observed. There was minimal type II pneumocyte hyperplasia, consistent with the early SARS-CoV-2 disease progression (4 dpi) (Figure 4A). All treatment groups exhibited decreased presence of interstitial pneumonia compared with the vehicle controls. MK-4482 treatments reduced interstitial pneumonia to 3 of 5 animals showing minimal lesions (Figure 4B). Treatment with PF-07321332 reduced the number of affected animals to 2 of 5, with both scoring minimal and mild in 1 lung lobe each (Figure 4C). Treatment with the drug combination reduced pneumonia even further, with only 1 of 5 animals displaying minimal lesions in 2 lung lobes (Figure 4D). Immunohistochemistry (IHC) of the same lung samples revealed a reduction in viral antigen between the vehicle group and all treated groups (Figure 4, E–H). Nasal turbinates were also collected at the time of euthanasia, with no observable pathology in either respiratory or olfactory epithelium (Supplemental Figure 2). Although there was an absence of pathology, IHC analysis did show scattered immunoreactivity in the vehicle animals and little to no observable viral antigen in treated groups (Supplemental Figure 2).

### Bioavailability of drugs in sera and lung.

To assess circulating drug levels and ensure bioavailability in the pulmonary compartment, levels of MK-4482, PF-07321332, and ritonavir were quantified in plasma collected prior to drug administration and in clarified lung homogenates collected at necropsy (Table 1). As previously described (21), EIDD-1931, the nucleobase metabolite of the MK-4482 prodrug, was used as a surrogate. MK-4482 signals were analyzed in all samples, and a standard curve was assessed. No signals above the limit of detection were observed because of rapid metabolism. Plasma was collected prior to dosing, and levels of each therapeutic molecule reflected the lowest circulating concentrations over the treatment course. At each time point, levels of all 3 drugs were readily detectable according to treatment group, with a mean of 46.47 nM for EIDD-1931 and 12.75 nM for PF-07321332 in plasma and 21.52 nmol/g for EIDD-1931 and 0.06 nmol/g for PF-07321332 in lungs for the single-therapy groups. Combination therapy resulted in similar concentrations of EIDD-1931 in the lung and plasma compared with the single therapy at 76.17 nM and 23.84 nmol/g, respectively. Levels of PF-07321332 were consistent in the plasma of combination therapy animals at 19.21 nM compared to single-therapy animals. Lung levels of PF-07321332 were elevated in combination therapy animals compared with single therapy at 0.36 nmol/g, suggesting a potential interaction between treatments leading to higher levels of PF-07321332 in the lung. All values agreed with the treatment scheme in both plasma and lung homogenate samples, with anticipated slight temporal fluctuations. Lung levels of EIDD-1931 were in good agreement with previous data examining efficacy of MK-4482 in the hamster model (21).

## Discussion

The continued emergence and selection of SARS-CoV-2 variants within populations with preexisting levels of spike-focused immunity remain a threat to the long-term effectiveness of current commercial spike-based SARS-CoV-2 vaccines and treatment approaches. It is therefore important to improve existing drugs/dosing regimens and to continue the development of new drugs that work independently from an effect on the spike protein. MK-4482 and PF-07321332 are compounds targeting the SARS-CoV-2 polymerase and protease, respectively (14, 15), and are obvious candidates. Both compounds have shown SARS-CoV-2 antiviral efficacy as single treatments in tissue culture (18, 21) and rodent and ferret models (21–25). Molnupiravir (MK-4482) and Paxlovid (PF-07321332 and ritonavir) have recently been approved for patients considered high risk for developing severe COVID-19 (11, 12).

Neither MK-4482 or PF-07321332 has yet been evaluated for antiviral efficacy in a SARS-CoV-2 nonhuman primate (NHP) model to our knowledge. Using the rhesus macaque model of SARS-CoV-2 infection (19), we assessed the antiviral activities of both compounds individually and in combination against the SARS-CoV-2 Delta VOC. The Delta VOC was selected as this variant has been shown to cause the most severe infection of all VOCs in rhesus macaques, especially compared with the rather mild infection by Omicron (26). Usage of the Delta VOC ensured use of the most stringent challenge model to measure treatment efficacy.

Individual treatment with both compounds showed a similar and significant reduction in SARS-CoV-2 viral loads in the upper and lower respiratory tract reducing SARS-CoV-2 shedding and replication, ultimately leading to less severe respiratory disease compared with vehicle-treated animals. Although both drugs reduced viral replication and disease, they did not completely absolve either. As the modes of action for MK-4482 and PF-07321332 are distinct, a combined therapy is potentially beneficial over monotherapy. This was supported by recent in vitro data demonstrating a synergistic antiviral effect of MK-4482 and PF-07321332 against Delta and Omicron VOCs in comparison with individual drug treatment (27, 28) and by in vivo data for a Korean strain isolated early in the pandemic (29) using a transgenic mouse model (30). In our present study, combined administration of MK-4482 and PF-07321332 treatment in the rhesus macaque model was well tolerated as indicated by clinical observation and blood chemistry/hematology analyses showing no indication for adverse reaction. Compared with individual drug treatments, combined therapy resulted in increased efficacy, with decreased SARS-CoV-2 shedding and replication early postinfection and milder disease.

Dosing for the study was allometrically based on the clinical treatment schedules currently approved for use for COVID-19. Molnupiravir (MK-4482) is prescribed as an 800 mg twice daily oral treatment (1,600 mg total) within 5 days of symptom onset (31). Similarly, nirmatrelvir is an oral treatment prescribed for twice daily oral treatment of 300 mg (600 mg total) with the addition of 100 mg ritonavir (200 mg total) (32). Each pharmaceutically active compound was assessed in plasma taken at each clinical exam point prior to dosing and in lung homogenates at necropsy to confirm its presence at the desired level and to ensure the absence of any unanticipated drug interaction between the molnupiravir and nirmatrelvir treatments. The levels of each active compound were not negatively affected by the combined treatment regimen.

Phase III clinical trials of molnupiravir indicate the drug is effective in preventing severe disease, with fewer adverse events documented in the treatment than the placebo group (10). Paxlovid is also an effective treatment for COVID-19 when administered within 5 days of symptom onset (13). However, recent reports of viral recrudescence following the cessation of molnupiravir and Paxlovid monotherapy in a subset of patients has raised the question of efficacy in relation to treatment length and/or dosage (33, 34). A recent single-patient case study indicates that rebound, at least for Paxlovid, may not correspond to the development of genetic resistance (35). Combination therapy of MK-4482 and PF-07321332 may counteract the “rebound effect” and enable maintained use of the relatively short 5-day treatment course. Loss of efficacy of a single-treatment approach due to viral escape is also a concern. In vitro studies using coronaviruses related to SARS-CoV-2 suggest that molnupiravir may have a high genetic barrier to development of drug resistance (36). However, recent studies passaging SARS-CoV-2 under suboptimal levels of nirmatrelvir have shown the development of resistance (37, 38). The use of a combination therapy, as established here, would be expected to reduce the possibility for viral escape. This has been established for treatment of other genetically mutable viruses, such as hepatitis C and human immunodeficiency virus (39, 40), which may even be more prone to viral escape because of their chronic nature of infection.

Limitations to our study need to be addressed in future work. First, because of the mild level of clinical disease in rhesus macaques following SARS-CoV-2 infection, it was not possible to assess the combination therapy’s effect against severe disease. This effect could be addressed in lethal rodent models or clinical trials. A suggestion of such efficacy against more severe disease is indicated in the recent study using a lethal transgenic COVID-19 mouse model (30). Second, we tested the efficacy of only the current human dose for monotherapy. Dose reduction might also be achievable in combination therapy and needs to be carefully designed and analyzed. Third, escape mutant development needs to be studied, especially given the recent studies suggesting the susceptibility of nirmatrelvir to resistance development in vitro (37, 38).

Our present study provides critical preclinical data in the NHP COVID-19 surrogate model demonstrating that molnupiravir and Paxlovid are most effective when used in combination. Notably, this improved efficacy occurred without noticeable toxicity in the NHP model. This study therefore strongly supports the potential for combined use of these drugs in patients with COVID-19 as a safe and improved therapeutic option. In addition to improving clinical outcomes, combination therapy may also enable a reduction in the dose of each individual drug, as well as slowing down drug resistance development. Clinical trials will be needed to confirm the benefit of the combination therapy over monotherapy, but this confirmation seems immediately possible as molnupiravir and Paxlovid are already approved for the treatment of patients with COVID-19.

## Methods

### Study design.

Male and female rhesus macaques (2–12 years of age) were obtained from the colony on Morgan Island, South Carolina, owned by the NIH National Institute of Allergy and Infectious Diseases (NIAID). The animals were divided into vehicle (*n* = 5) or treatment (*n* = 5 for MK-4482, *n* = 5 for PF-07321332, and *n* = 5 for the MK-4482/PF-07321332 combination) groups prior to infection. MK-4482 (DC Chemicals), PF-07321332 (DC Chemicals), and ritonavir (DC Chemicals) were first dissolved in DMSO and then resuspended to 5 mL total volume in food-grade peanut oil for oral delivery. Treated rhesus macaques received 130 mg/kg MK-4482, 20 mg/kg PF-07321332 + 6.5 mg/kg ritonavir, or the combination of all 3 compounds (130 mg/kg MK-4482 + 20 mg/kg PF-07321332 + 6.5 mg ritonavir) every 12 hours beginning 12 hours postinfection (hpi) and ending 84 hpi. Vehicle controls received similar volumes of peanut oil (5 mL) on the same schedule. Animals were infected with a total of 2 × 10^6^ TCID_50_ of the SARS-CoV-2 Delta VOC by intranasal and intratracheal routes. Intranasal inoculation consisted of a 0.5 mL injection directly into each nare (1.0 mL total) with a Mucosal Atomization Device system (Teleflex). Intratracheal inoculations were performed with the use of a bronchoscope for deposition of 4 mL of virus directly into the main stem bronchi. All procedures were performed on anesthetized animals. Animals were monitored twice daily and scored blindly every morning by the same person for disease signs and progression as previously reported (19). Clinical exams were performed prior to challenge and at 0, 1, 2, and 4 dpi. Oropharyngeal, nasal, and rectal swabs, as well as whole blood and serum, were collected at every exam. BAL samples were collected at 1, 2, and 4 dpi. Animals were euthanized on 4 dpi and tissues were collected at necropsy.

### Virus and cells.

SARS-CoV-2 variant hCoV-19/USA/KY-CDC-2-4242084/2021 (B.1.617.2, Delta) was contributed by B. Zhou, N. Thornburg, and S. Tong (CDC, Atlanta, Georgia, USA). The viral stock was sequenced via Illumina-based deep sequencing to confirm identity and any possible contaminants. Virus propagation was performed in DMEM (MilliporeSigma) supplemented with 2% fetal bovine serum (Gibco), 1 mM l-glutamine (Gibco), 50 U/mL penicillin, and 50 μg/mL streptomycin (Gibco). Vero E6 cells, provided by R. Baric, University of North Carolina, Chapel Hill, North Carolina, USA, were maintained in DMEM (MilliporeSigma) supplemented with 10% fetal calf serum, 1 mM l-glutamine, 50 U/mL penicillin, and 50 μg/mL streptomycin.

### Viral genome detection.

A QIAamp Viral RNA Kit (QIAGEN) was used to extract RNA from swabs or tissue samples. A QuantiFast kit (QIAGEN) was used according to manufacturer’s protocols, and 1-step real-time RT-PCR was used to quantify subgenomic viral RNA using a region of the E gene (20). RNA standards counted by droplet digital PCR were used in 10-fold serial dilutions and run in parallel to calculate viral RNA copies.

### Virus titration assay.

Tissues were homogenized in 1 mL DMEM using a TissueLyzer (QIAGEN) and clarified by low-speed centrifugation (6,000*g* for 10 minutes at room temperature). Vero E6 cells were inoculated with 10-fold serial dilutions of homogenized lung or swab samples in 100 μL DMEM supplemented with 2% fetal bovine serum, 1 mM l-glutamine, 50 U/mL penicillin, and 50 μg/mL streptomycin. Cells were incubated for 6 days and then scored for cytopathogenic effects, and TCID_50_ was calculated via the Reed-Muench formula (41).

### Hematology and serum chemistry.

Hematologic analysis was completed on a ProCyte DX (IDEXX Laboratories). Red blood cells, hemoglobin, hematocrit, mean corpuscular volume, mean corpuscular hemoglobin, mean corpuscular hemoglobin concentration, red cell distribution weight, platelets, mean platelet volume, white blood cells, neutrophil count (absolute number and percentage), lymphocyte count (absolute number and percentage), monocyte count (absolute number and percentage), eosinophil count (absolute number and percentage), and basophil count (absolute number and percentage) were all evaluated. Serum chemistries were analyzed on a VetScan VS2 Chemistry Analyzer (Abaxis). The following parameters were evaluated: glucose, blood urea nitrogen, creatinine, calcium, albumin, total protein, alanine aminotransferase, aspartate aminotransferase, alkaline phosphatase, total bilirubin, globulin, sodium, potassium, chloride, and total carbon dioxide.

### Pharmacokinetics.

All solvents and extraction reagents were liquid chromatography-mass spectrometry grade and purchased from Thermo Fisher Scientific. Lung sections were extracted and levels of EIDD-1931 were measured as previously described (21). Plasma samples were processed via dilution of 100 μL of plasma into 300 μL of ice-cold methanol. The precipitant was cleared by centrifugation at 6,000*g* for 10 minutes at 4°C, and the supernatant was prepared for sample injection. Multiple reaction monitoring (MRM) ion pair signals were developed and optimized for ritonavir and PF-07321332 (nirmatrelvir) from standards (Supplemental Table 1). All pharmaceutical actives were measured from a single injection on a Sciex ExionLC AC system with a Waters XBridge Amide column (130 Å, 3.5 μm, 3 mm × 100 mm) with binary gradient elution from 95% acetonitrile, 0.8% acetic acid, 10 mM ammonium acetate to 50% acetonitrile, 0.8% acetic acid, 10 mM ammonium. All signals were detected in positive mode using a 5500 QTRAP mass spectrometer (AB Sciex) equipped with an electrospray ionization source (curtain gas: 40, collisionally activated dissociation gas: med, ion-spray voltage: 2,500, temperature: 45°C, ion source gas 1: 50, ion source gas 2: 50) and compared to an 8-point standard curve. All molecules of interest were detected using 2 MRM pairs and quantified using the higher signal-to-noise MRM. Limit of quantification was 400 pg/mL for PF-07321332 and 300 pg/mL for ritonavir.

### Histopathology.

Tissue samples were collected, fixed in 10% formalin for 7 days, and processed with a Sakura VIP-6 Tissue Tek, on a 12-hour automated schedule, using a graded series of ethanol, xylene, and PureAffin. Samples were embedded in PureAffin polymer (Cancer Diagnostics), sectioned at 5 μm, and dried overnight at 42°C prior to H&E staining. For IHC, tissues were processed using the Discovery Ultra-automated stainer (Ventana Medical Systems) with a ChromoMap DAB kit (Roche Tissue Diagnostics catalog 760-159). Specific immunoreactivity was detected using a validated GenScript, U864YFA140-4/CB2093 NP-1 SARS-CoV-2-specific antiserum (1:1,000 dilution), and a secondary anti-rabbit IgG polymer (catalog MP-6401) from Vector Laboratories ImPress VR as previously described (21).

### Statistics.

Statistical analysis was performed in GraphPad Prism 9. Difference in viral load and infectious titers between study groups was assessed by ordinary 2-way ANOVA (multiple time points) or by multiple comparisons using the Kruskal-Wallis test (single time points). AUC was calculated and then analyzed by an ordinary 1-way ANOVA. *P* values of less than 0.05 were considered statistically significant.

### Study approval.

SARS-CoV-2 studies were approved by the Institutional Biosafety Committee (IBC) and performed in the high biocontainment laboratories at Rocky Mountain Laboratories (RML), NIAID, NIH. IBC-approved standard operating procedures were used for sample removal. The RML IACUC approved the animal study protocol. Studies followed institutional guidelines for animal use and the guidelines and basic principles in the NIH *Guide for the Care and Use of Laboratory Animals* (National Academies Press, 2011), the Animal Welfare Act, the US Department of Agriculture, and the US Public Health Service Commissioned Corps Policy on Humane Care and Use of Laboratory Animals. Rhesus macaques were singly housed in adjacent cages to enable social interactions. The animal room was climate-controlled with a fixed 12-hour light/12-hour dark cycle. Animals were provided commercial chow twice daily with vegetables, fruit, and treats to supplement. Water was available ad libitum. Human interaction, manipulanda, toys, videos, and music were provided for enrichment.

## Author contributions

KR, MAJ, and HF contributed to the design, execution, and writing of the manuscript. KR, AO, FF, JC, JL, MCL, WFB, EB, BS, CMB, MAJ, and HF contributed experimental support and data analysis. CS and GS contributed to the pathological analysis. HF secured funding for the study.

## Supplementary Material

Supplemental data

## Figures and Tables

**Table 1:**
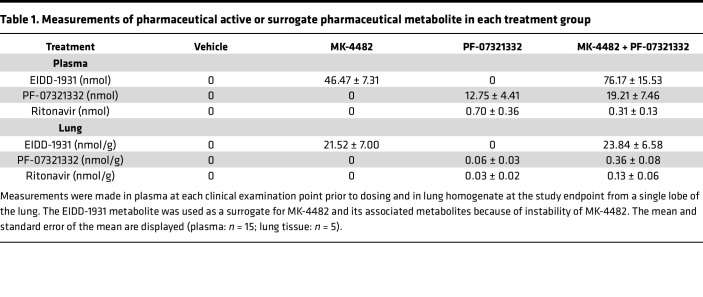
Measurements of pharmaceutical active or surrogate pharmaceutical metabolite in each treatment group

## Version 1 12/27/2022

In-Press Preview

## Version 2 02/22/2023

Electronic publication
