# Supplementary material for: Combined molnupiravir-nirmatrelvir treatment improves the inhibitory effect on SARS-CoV-2 in macaques
Source: JCI Insight. 2023 Feb 22;8(4):e166485. doi: 10.1172/jci.insight.166485 (PMC9977490; doi:10.1172/jci.insight.166485)
Supplement: Supplemental data [file jciinsight-8-166485-s130.pdf]

SUPPLEMENTARY MATERIALS

Title: Combined Molnupiravir and Nirmatrelvir Treatment Improves the Inhibitory Effect on SARS-CoV-2 in Rhesus Macaques

Authors: Kyle Rosenke[1], Matt C. Lewis[1], Friederike Feldmann[2], Eric Bohrnsen[3], Benjamin Schwarz[3], Atsushi Okumura[1], W. Forrest Bohler[1], Julie Callison[1], Carl Shaia[2], Catharine M. Bosio[3], Jamie Lovaglio[2], Greg Saturday[2], Michael A. Jarvis[1,4,5], Heinz Feldmann[1†]

Affiliation: [1]Laboratory of Virology, [2]Rocky Mountain Veterinary Branch and [3]Laboratory of Bacteriology, Division of Intramural Research, National Institute of Allergy and Infectious Diseases, National Institutes of Health, Hamilton, MT, USA; [4]University of Plymouth, Plymouth, Devon, UK; [5]The Vaccine Group Ltd, Plymouth, Devon, UK

†Corresponding author: Heinz Feldmann, Rocky Mountain Laboratories, 903 S 4th Street, Hamilton, MT, US-59840; Tel: (406)-375-7410; Email: feldmannh@niaid.nih.gov

One Sentence Summary: Molnupiravir and nirmatrelvir treatment of SARS-CoV-2 is most effective in the rhesus macaque COVID-19 model when used in combination.

Supplementary Table 1

Target	MRM pair (m/z)	DP (V)	EP (V)	CE (V)	CXP (V)
PF-07321332*	500.0/110.0	75	10	30	7
PF-07321332	500.0/69.0	180	5	80	30
Ritonavir*	721.0/140.0	185	15	85	20
Ritonavir	721.0/268.0	120	5	30	40

*Used for quantification

Supplementary Table 1: MRM signals were identified and optimized for ritonavir and PF-07321332 (nirmatrelvir) from standards.Key: MRM: multiple reaction monitoring; DP: declustering potential; EP: entrance potential; CE: collision cell entrance potential; CXP: collision cell exit potential

Figure S1

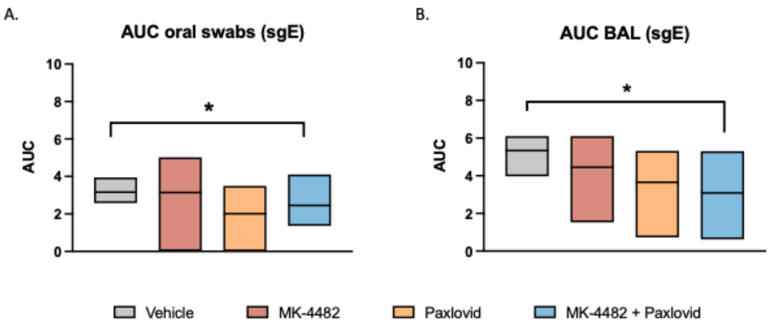

Supplementary Figure 1. AUC analysis of oral swabs and BAL fluid. Viral RNA loads from oral swabs **(A)** and BAL **(B)** samples were determined by quantitative RT-PCR targeting sgE RNA as a surrogate for replication and shedding. Copy numbers of viral genomes were calculated for each animal per day, AUC was then calculated over the course of the study and displayed in a boxplot with the mean displayed. Ordinary one-way ANOVA with multiple comparisons were used to evaluate significance (*P-value = 0.01 to 0.05).

Figure S2

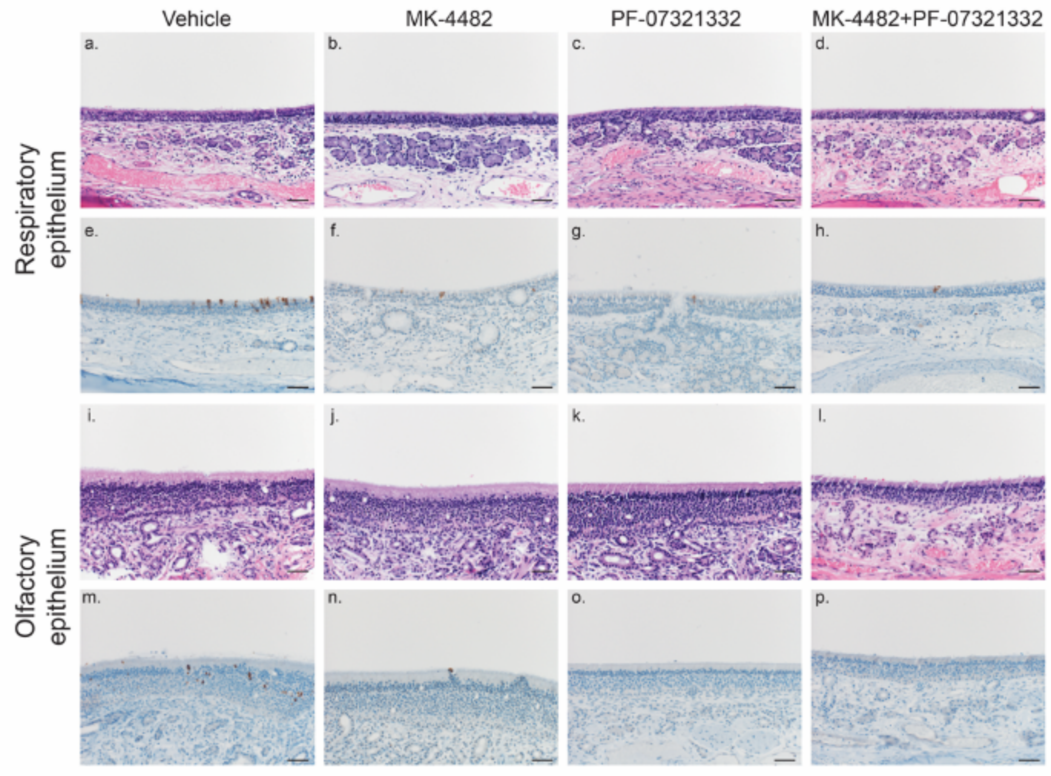

Supplementary Figure 2. Combination therapy reduced antigen load in olfactory and respiratory epithelium from nasal turbinates. Tissues were collected on 4dpi and stained with H&E or IHC for analysis. **H&E staining of representative tissues sections of the respiratory epithelium (A-D).** No pathology was found in H&E stains of the nasal turbinates. **IHC staining of SARS-CoV-2 antigen in respiratory epithelium (E-H).** Reduced or no IHC stain was found in the respiratory epithelium of MK-4482, PF-07321332 and MK-4482 + PF-07321332 treated animals compared to vehicle controls. **H&E staining of representative tissues sections of the olfactory epithelium from nasal turbinate (I-L).** No pathology was found in the olfactory epithelium. **IHC staining of SARS-CoV-2 antigen in olfactory epithelium (M-P).** IHC analysis did show scattered immunoreactivity in the vehicle treated animals and little to no

observable viral antigen in the MK-4482, PF-07321332 and MK-4482 + PF-07321332 treated animals 200X, Bar=50µm
